# Insights from farming *Macrocystis pyrifera* offshore: phenotypic analysis, genome-wide association studies, genomic selection

**DOI:** 10.1038/s41437-026-00855-4

**Published:** 2026-06-18

**Authors:** Maxim Kovalev, Gabriel Montecinos Arismendi, Christie Yorke, Rachael M. Wade, Gary Molano, Robert Miller, Daniel Reed, Filipe Alberto, Sergey Nuzhdin

**Affiliations:** 1 Department of Biological Sciences, University of Southern California, Los Angeles, CA USA; 2 Department of Biological Sciences, University of Wisconsin-Milwaukee, Milwaukee, WI USA; 3 Hortimare, Heerhugowaard, The Netherlands; 4 Marine Science Institute, University of California, Santa Barbara, CA USA; 5 Department of Biological Sciences, California State Polytechnic University, Humboldt, CA USA; 6 Kelp Ark, San Pedro, CA USA

**Keywords:** Genome-wide association studies, Genetic markers, Quantitative trait

## Abstract

Seaweed farming, as a part of aquaculture, offers a sustainable alternative to modern agricultural practices; however, genetic enhancement and breeding programs for most species are underdeveloped. We aimed to advance seaweed domestication by focusing on giant kelp (*Macrocystis pyrifera*), the fastest-growing haplodiplontic brown alga, which has significant ecological and commercial importance. We analyzed phenotypic data from two offshore experimental farms conducted in 2019 and 2020, which involved hundreds of outplanted genetically diverse sporophytes. We found that outplanting season and farm design had significant effects on giant kelp biomass. Broad-sense heritability estimates showed moderate (0.27-0.50) genetic contributions to two phenotypes, carbon content and total biomass. Genome-wide association studies for these phenotypes resulted in three statistically significant SNPs, located near or within genes involved in carbohydrate metabolism and cytoskeletal functions. In addition, we applied genomic selection models that integrated sporophyte phenotypes and parental gametophyte genotypes. These models utilized reduced sets of GWAS-ranked SNPs obtained by a procedure based on linkage disequilibrium estimations. Model testing yielded cross-validation accuracy values of up to 0.84 and predictive accuracy values of up to 0.40, demonstrating the potential of marker-assisted breeding for phenotype improvement. Our results provide foundational genomic resources and tools for domesticating and breeding *M. pyrifera*, forming a basis for developing giant kelp varieties with desirable traits.

## Introduction

Modern agriculture is notorious for excessive land use, water pollution, and biodiversity loss. At the same time, climate change is reducing crop yields and exacerbating food insecurity through altered weather patterns, rising temperatures, and extreme climatic events (Foley et al. 2011; Wheeler and von Braun 2013; Tilman et al. 2017). In this context, aquaculture has grown rapidly as an alternative in the food industry, with seaweed farming offering numerous sustainability benefits and aiding in climate change mitigation through innovation and responsible production (Duarte et al. 2017, 2022; Gentry et al. 2017; FAO 2022).

Despite its advantages, seaweed aquaculture remains constrained by taxonomy and geography. As of 2020, eight species made up the majority of global production; Asia led with 99.54% of the output, while North America contributed the least (less than 0.001%) (FAO 2022). Nevertheless, seaweed aquaculture in North America, and especially in the United States, is rapidly expanding. Consumer interest in new proteins, health supplements, sustainable additives, and food security has led to more farms and funding opportunities in the US since 2010 (Kim et al. 2019). Identifying regionally appropriate species with high ecological and commercial value is, therefore, a critical step toward expanding seaweed aquaculture.

Among the widely distributed seaweeds along the North American coasts, *Macrocystis pyrifera* (Phaeophyceae, Laminariales) stands out for several reasons. Also known as giant kelp, *M. pyrifera* is one of the fastest-growing organisms and a foundation species that provides critical ecosystem services, including carbon sequestration and wave energy buffering (Castorani et al. 2018; Miller et al. 2018; Rassweiler et al. 2018; Lamy et al. 2020; Eger et al. 2023). This brown alga has a heteromorphic haplodiplontic life cycle with macroscopic diploid (sporophyte) and microscopic haploid (gametophyte) stages, making it suitable for germplasm biobanking (Wade et al. 2020). Giant kelp biomass is mostly wild-harvested (Purcell-Meyerink et al. 2021) and used as a source of alginate and as a raw material for various products, including pharmaceuticals, fertilizers, and biofuels (Camus et al. 2016; Chopin and Tacon 2021; Mollah et al. 2021). With the highest genetic diversity reported in Southern California (Klingbeil et al. 2022), giant kelp is a promising candidate for domestication and cultivation in the United States, given its ecological importance and economic potential.

The establishment of genetic resources and application of genetics-based selection methods can significantly facilitate and accelerate breeding programs (Jannink et al. 2010; Bhat et al. 2016; Budhlakoti et al. 2022). Nevertheless, genomic selection (GS) studies have been limited to *Saccharina latissima* (Huang et al. 2023; Bråtelund et al. 2026). In *M. pyrifera*, prior genetic research has largely focused on population structure using microsatellites or mitochondrial markers (Alberto et al. 2010, 2011; Macaya and Zuccarello 2010; Johansson et al. 2015), as well as transcriptomic analyses based on de novo assemblies (Konotchick et al. 2013; Salavarría et al. 2018; Lipinska et al. 2019; Paul et al. 2020). The recent publication of two reference genomes (Paul et al. 2022; Diesel et al. 2023), including a highly complete and annotated assembly, now enables fine-scale population genomics and association studies (Bemmels et al. 2025).

The objective of this study was to develop genomic resources to support the domestication of giant kelp for commercial production in Southern California. Using two years (2019-2020) of phenotypic data from experimental offshore farms, we conducted genome-wide association studies (GWAS) to identify genetic variants associated with traits relevant to cultivation and breeding. We further developed a genomic selection model that combines sporophyte phenotypic and gametophyte genotypic data to evaluate the predictive ability for selected traits. Finally, we integrated GWAS results with genomic selection to assess how subsets of trait-associated SNPs influence prediction accuracy.

## Methods

### Gametophyte isolation, DNA sequencing, and SNP calling

Obtaining gametophyte collection, performing DNA extraction, and sequencing are reported in Osborne et al. (2023) and briefly described here. First, to isolate gametophytes, reproductive blades of *Macrocystis pyrifera* (sporophylls) were collected from four geographically and genetically different locations in Southern California in December 2018. These regions are Arroyo Quemado (AQ), Catalina Island (CI), Camp Pendleton (CP), and Leo Carrillo (LC). The sporophylls were shipped overnight to the University of Wisconsin-Milwaukee, where spore release was performed according to the Oppliger method (Oppliger et al. 2011) approximately 24 h after collection. Spores were settled onto Petri dishes (in two dilutions of 10 and 100 spores per mm^2^) and placed in growth chambers (12 °C; red light, 20 μmol photons m^−2^s^−1^; 12:12 h Light:Dark photoperiod) to germinate into gametophytes with PES medium (Provasoli 1968) replaced every five weeks while the spores were in this low-density phase. Once reaching 100 μm in size, the gametophytes were individually isolated by sex and vegetatively grown under the same conditions with increased light intensity (30 μmol photons m^−2^s^−1^) to facilitate faster growth. The PES medium was replaced every week. After reaching 2 mm in diameter, clones from gametophyte cultures were selected and fragmented mechanically to promote exponential biomass growth.

Before DNA extraction, 50–100 mg of gametophyte tissue biomass was obtained by centrifuging and discarding the supernatant for each gametophyte culture. The biomass was then pulverized using liquid nitrogen, and the NucleoSpin 96 Plant Kit (Macherey-Nagel) was used to extract high-quality genomic DNA. Library preparation and sequencing were performed by the BGI North America NGS lab (Illumina S4 Novaseq platform; 150 bp paired-end reads), generating ~11.2 GB of genomic data (87 million reads) per sample.

A detailed description of sequencing quality assessment, SNP calling, and SNP processing can be found in Harden et al. (2024). Briefly, raw reads were processed to remove adapters and low-quality sequences, followed by mapping to the *M. pyrifera* nuclear genome (Diesel et al. 2023). Our samples contained substantial non-host DNA due to co-extraction with associated microbiota, resulting in a low average mapping rate (~20%) and an effective host genome coverage of ~4-5x per sample. Duplicate reads were marked, and genetic variants were called with a ploidy of 1 using GATK4 (Van der Auwera and O’Connor 2020). The resulting VCF was filtered to retain high-quality biallelic SNPs based on scaffold presence, mean depth, and missing data using vcftools (Danecek et al. 2011), ensuring robust genotype calls in haploid samples and minimizing bias in downstream association analyses. The final dataset comprised 504 gametophytes (90 male and 414 female) and 1,515,399 biallelic SNPs. Missing data were imputed using an algorithm described in Harden et al. (2024), which uses LinkImpute (Money et al. 2015). SNP data were coded binary based on the absence or presence of a minor allele.

### Sporophyte farm design and phenotyping

The full description of the farm design and sporophyte preparation can be found in Osborne et al. (2023). Here we describe the main features. Before seeding on polyvinyl lines (2 mm diameter, 6 cm long), gametophytes were fragmented into 5–10 cell-long filaments using a pellet pestle. Female gametophytes were seeded a day earlier than male ones, followed by gradual exposure to increasing white light intensities (15, 22, 35, 60 μmol photons m^−2^s^−1^) over four days. After the crosses developed into sporophytes, the polyvinyl lines were attached to nylon seeding lines (25 m long, 1/4 in diameter) at 0.5 m intervals, resulting in 50 sporophyte genotypes on each seeding line. During outplanting, divers zip-tied the seedling lines to moored long lines (1 in diameter, 137 m long). Each long line had five seeding lines with 250 sporophyte genotypes in total.

The crossing scheme for 2019 was directed to explore most of the genetic variation under limited conditions. Since females dominate in our collection (100 males vs. 500 females), we varied genetic diversity only through them. We crossed all female gametophyte genotypes with one randomly selected male gametophyte genotype of LC origin in five replicates, resulting in a total of 2500 seeded polyvinyl lines. These polyvinyl lines were outplanted to ten moored long lines placed as five pairs of two adjacent lines, with each pair containing one replicate of the 500 unique sporophyte genotypes. The spatial positioning of the longline pairs was designed to minimize location effects on phenotypic expression (Osborne et al. 2023). Seeded lines were outplanted to the experimental farm in May 2019 and harvested for phenotypic measurements in September 2019.

The 2020 crossing design aimed to measure repeatability in the phenotypic variation observed in 2019. The number of genotypes was decreased to 96, while the number of replicates was increased to 10. The 2020 crosses were chosen based on the 2019 data. Namely, three random females and three females that contributed to the three highest-biomass sporophyte genotypes with the lowest-replicate variation were selected from each site (24 female gametophyte genotypes in total). These females were crossed with four males, one per site. The AQ, CP, and CI males were randomly selected, while the LC male remained the same. The resulting 960 sporophytes (96 sporophyte genotypes x 10 replicates) were outplanted to four moored longlines on the experimental farm in March 2020 and harvested in July 2020. Visual representations of the crossing schemes are depicted in Supplementary Fig. 1.

Due to permit limits and resource constraints, we measured only non-reproductive, low-cost traits that are nevertheless important to farmers and industry. These included quantifying survivorship for each sporophyte genotype, counting the number of clonemate sporophytes growing on each polyvinyl line, total wet mass of blades and stipes per replicated sporophyte genotypes. Total biomass was estimated as the sum of the blades and stipes’ mass. For 2019, a portion of sporophytes was analyzed for carbon content and nitrogen content using elemental analyzer and muffle furnace combustion in the Soil and Forage Analysis laboratory at the University of Wisconsin.

Populations of gametophytes, cross-populations, replicate ID, long line ID, seedling line ID, dates of outplanting and harvesting were recorded as environmental variables. The aliases for the phenotypes and environmental variables are provided in Supplementary Table 1.

### Analysis of phenotypic data

For each farm year, we constructed correlation matrices (Pearson’s correlation) exploring the phenotypes. At the same time, for the environmental variables, we used the uncertainty coefficient (UC) as a proxy for correlation since the variables are categorical. For variables X and Y, ${UC}\left(X,Y\right)$ tells what portion of X can be predicted given Y, meaning that ${UC}\left(X,Y\right)\ne {UC}\left(Y,X\right)$. We focused on total biomass (Mass.Total), carbon content (Carbon), nitrogen content (Nitrogen) from the recorded phenotypes and selected cross-population (Pop.Cross), long line ID (Line.Long), seeding line ID (Line.Seed) from the environmental variables.

The association between sporophyte survivability and the selected environmental variables, including sporophyte genotypes, was tested using the Chi-squared test of independence. We separately studied how the number of clonemate sporophytes on a polyvinyl line (individual counts; Count.Ind) affected the phenotypes by organizing them into 14 groups (individual counts = 1, = 2, = 3, …, = 13, >13) and approximating trends. We then used the Kruskal-Wallis test to examine whether sporophyte genotypes, cross-population, long line ID, seeding line ID, and individual counts affect variation in the phenotypes.

Using the phenotypes affected by genotypic variation, we estimated the best linear unbiased estimations (BLUEs) representing the genetic contribution to phenotypes independent of environmental effects. To achieve this, we fitted LMM models with effects previously determined to be statistically significant by the Kruskal-Wallis test, using genotype as a fixed effect term and excluding cross-population, as we are directly addressing population stratification in the association studies. We also fitted these models setting genotype as a random effect, which allowed us to obtain the best linear unbiased predictors (BLUPs) for the genotype term and estimate Holland’s, the regression-based, and the shrinkage-based versions of broad-sense heritability (H^2^), defined in Schmidt et al. (2019). To fit LMM models and extract BLUEs and BLUPs, we used the R packages lme4 (Bates et al. 2015) and emmeans (Lenth 2025).

### Genome-wide association studies

To investigate associations between sporophytes’ genotypes and phenotypic data represented by BLUEs, we applied several genome-wide association models to the 2019 farm data. These models are general linear model (GLM), mixed linear model (MLM), FarmCPU (Liu et al. 2016), and Blink (Huang et al. 2019). All are implemented in the GAPIT R package (Wang and Zhang 2021).

Regarding sporophyte SNP data, we recreated the genotypes of the harvested sporophytes by leveraging the features of the giant kelp life cycle. Namely, since meiosis and recombination occur before spore release (Reed 1990; Le et al. 2022), sporophytes inherit one set of chromosomes from each parental gametophyte, resulting in diploid organisms with “stacked” or combined genotypes. Computationally, this means that a sporophyte’s SNP is the sum of the parental gametophytes’ SNPs when coded from the perspective of the number of minor alleles. From the genomic data, we recovered 407 and 285 sporophyte genomes that had associated BLUEs for total biomass and carbon content, respectively. For the analysis, we kept only SNPs with MAF > 0.05. To account for population stratification, we performed principal component analyses on these datasets. using the SNPRelate R package (Zheng et al. 2012). Each GWAS model was fitted with a different number of principal components (none, 1, 2, …, 6).

Overall, we fitted 28 GWAS models for each phenotype. We assessed the models both qualitatively and quantitatively. The former was performed by evaluating quantile-quantile (QQ) plots and the number of statistically significant SNPs based on the Benjamini-Hochberg threshold. The latter was done by estimating the genomic inflation factor (Lambda). Genes containing statistically significant loci or located within 10 Kb in both directions of the significant loci were considered putative genes affecting phenotypic variation.

Gene Ontology (GO) (Ashburner et al. 2000; The Gene Ontology Consortium et al. 2023) and Eukaryotic Orthologous Groups (KOG) (Tatusov et al. 1997, 2003; Galperin et al. 2025) annotations were obtained from the *Macrocystis pyrifera* JGI genome portal (https://phycocosm.jgi.doe.gov/Macpyr2/Macpyr2.home.html). To annotate SNP effects, we used the SnpEff software (Cingolani et al. 2012).

### Genomic selection models

Genomic selection (GS) models were built using the rrBLUP R package (Endelman 2011; Endelman and Jannink 2012). The main feature of the models is that genomic estimated breeding values (GEBVs) are obtained for gametophytes based on data from sporophytes. For that, we designed an underlying LMM as follows

$$\begin{array}{ccc}y=X\beta +{Zu}+\varepsilon , & u \sim {MV}{N}_{m+f}\left(0,{\sigma }_{a}^{2}K\right), & \varepsilon \sim {MV}{N}_{n}\left(0,{\sigma }_{e}^{2}{I}_{n}\right),\end{array}$$

$$\begin{array}{ccc}Z=0.5\left[\begin{array}{cc}{Z}_{{male}} & {Z}_{{female}}\end{array}\right],\, & {Z}_{{male}}=\left[\begin{array}{c}\begin{array}{c}{o}_{m}\left({i}_{1}\right)\\ {o}_{m}\left({i}_{2}\right)\end{array}\\ \begin{array}{c}\ldots \\ {o}_{m}\left({i}_{n}\right)\end{array}\end{array}\right], & {Z}_{{female}}=\left[\begin{array}{c}\begin{array}{c}{o}_{f}\left({j}_{1}\right)\\ {o}_{f}\left({j}_{2}\right)\end{array}\\ \begin{array}{c}\ldots \\ {o}_{f}\left({j}_{n}\right)\end{array}\end{array}\right],\end{array}$$

where n is the number of sporophytes; m and f are the total numbers of male and female gametophytes, respectively, crossed to produce the sporophytes; y in is an n-vector of phenotype values; X is an $n\times \left(p+1\right)$ matrix of covariates including p principal components and a column of 1 s; β is a $\left(p+1\right)$-vector of the corresponding coefficients including the intercept; Z is an $n\times \left(m+f\right)$ design block matrix represented by two parts, ${Z}_{{male}}$ and ${Z}_{{female}}$; ${o}_{N}\left(i\right)$ is an N-row where the i-th element equals 1, and the others are 0; ${i}_{1},{i}_{2},\ldots ,{i}_{n}$ are the indexes of male gametophytes used to produce sporophyte $\mathrm{1,2},\ldots ,n$, respectively; ${j}_{1},{j}_{2},\ldots ,{j}_{n}$ are the indexes of female gametophytes used to produce sporophyte $\mathrm{1,2},\ldots ,n$, respectively; u is an $\left(m+f\right)$-vector of random effects obeying ${MV}{N}_{m+f}\left(0,{\sigma }_{a}^{2}K\right)$, a multivariate normal distribution with a 0 mean vector and a covariance matrix ${\sigma }_{a}^{2}K$; ${\sigma }_{a}^{2}$ is a variance of random effects; K is an $\left(m+f\right)\times \left(m+f\right)$ kinship matrix; ε is an n-vector of errors obeying ${MV}{N}_{n}\left(0,{\sigma }_{e}^{2}{I}_{n}\right)$; ${\sigma }_{e}^{2}$ is a variance of the residual errors; ${I}_{n}$ is an n×n identity matrix.

To examine the predictability of the phenotypes (BLUEs), we performed a cross-validation procedure using the 2019 data. The procedure consisted of 10,000 repetitions, where one repetition included several steps: (1) selecting 75% random sporophytes from each cross-population, (2) identifying their parental gametophytes, (3) fitting the LMM and extracting GEBVs for the parental gametophytes, (4) predicting GEBVs for the remaining gametophytes according to Clark and van der Werf (2013), (5) computing genetic additive effects (GAEs) for the remaining 25% of sporophytes as an average between male and female gametophytes’ GEBVs, (6) estimate prediction accuracy, which is Spearman correlation between the GAEs and the sporophytes’ phenotype values. Additionally, we did a separate test in which we predicted total biomass from the 2020 data using the 2019 data. For that, we identified shared sporophytes’ genotypes between the two datasets, filtered them out from the 2019 data (22 out of 407), repeated steps (2)-(4), estimated GAEs for the 2020 total biomass, and calculated Spearman correlation.

To optimize the prediction accuracy, we ran these procedures for different sets of SNPs used to obtain the kinship matrix. Specifically, for each phenotype, we performed chromosome-wide LD clumping. This involved hierarchical clustering based on LD (*r*^2^) between SNPs (*r*^2^ > 0.1) and selecting the SNP with the lowest GWAS *p*-value in each cluster as a representative. The two obtained sets were sorted by the *p*-values and used to create subsets based on ranks (for example, the top 100, top 500, top 1000, etc.). This approach was reported to be successful in multiple studies (Kaler et al. 2022; Yan et al. 2023).

## Results

### Description and analysis of farm data

We performed correlation analysis on the recorded phenotypes and environmental variables, the descriptions of which are provided in Supplementary Table 1. Cross-population (Pop.Cross) was found to be independent of other environmental variables except male and female gametophyte populations, as expected. Long line ID (Line.Long) and seeding line ID (Line.Seed) encompassed information about the remaining variables, which aligns with the non-random design of outplanting and harvesting (Supplementary Fig. 2). Regarding the phenotypes, all the measured mass values were positively correlated with total biomass (Mass.Total), which is their sum. In the 2019 farm, correlations among total biomass, carbon content (Carbon), and nitrogen content (Nitrogen) varied from 0.16 to 0.19, all of which were statistically significant (*p* ≤ 0.0001). Individual counts (Count.Ind) and total biomass were positively correlated, with Pearson’s coefficients of 0.27 and 0.55 for 2019 and 2020, respectively (*p* ≤ 0.0001). The correlation between individual counts and nitrogen content was 0.089 (*p* ≤ 0.01), whereas for carbon content, it was -0.019 (*p* > 0.05) (Supplementary Fig. 3).

Sporophytes on 1,686 of the 2,500 lines (67.44%) outplanted in 2019 survived to be harvested, whereas sporophytes on 627 of 960 (65.31%) lines outplanted in 2020 survived to be harvested. Of those that survived, 1669 and 614 records were analyzed for total biomass in 2019 and 2020, respectively, 1269 records for carbon content, and 1,267 records for nitrogen content in 2019 (Supplementary Table 2). We used the chi-squared test of independence to analyze relationships between survivability in each year and factors such as sporophyte genotype, cross-populations, long line ID, and seeding line ID. As a result, we observed only one instance of independence, long line ID from the 2020 data (*χ*^2^(3, 960) = 2.484, *p* = 0.478), while the other cases showed statistically significant dependence (*p* ≤ 0.0001); all are reported in Supplementary Table 3.

**Fig. 1:**
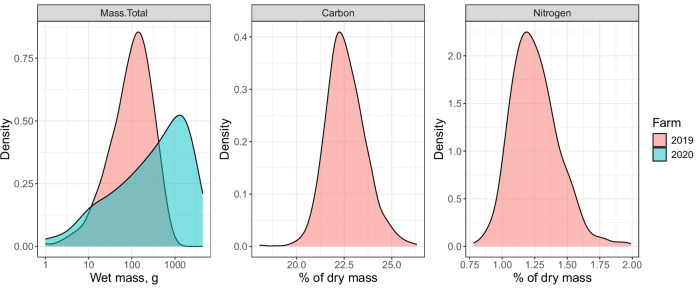
Distributions of the phenotypes from the 2019 and 2020 farm data. Mass.Total – total biomass; Carbon – carbon content; Nitrogen – nitrogen content.

In this study, we aimed to analyze three phenotypes: total biomass, carbon content, and nitrogen content. Their distributions are shown in Fig. 1. Total biomass (wet mass) ranged between 1 and 1002 g in 2019 and between 1 and 4414 g in 2020. Sporophytes reaching a total biomass of 1 g were rare and belonged to a single genotype (*n* = 5 in 2019; *n* = 8 in 2020), with no two replicates exhibiting this mass. Carbon content constituted between 18.05 and 26.32% of dry mass, while the nitrogen content ranged between 0.78 and 1.99% of dry mass. Comparing total biomass between the two years, the median for 2019 was 110 g, whereas the median for 2020 was 405 g.

**Fig. 2:**
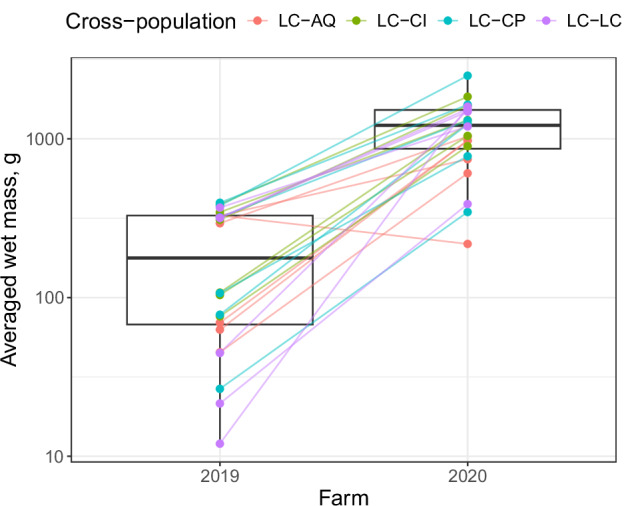
Performance comparison of the crosses shared between the two farms (*n* = 24).

Twenty-four genotypes were shared between the two years. We performed non-parametric two-way ANOVA (Wobbrock et al. 2011; Kay et al. 2025) to analyze the effect of genotype and year on total biomass. The analysis revealed that there was no statistically significant interaction between the genotype and year effects (*F*(23, 233) = 1.4237, *p* = 0.1). However, the main effects of genotype and year were statistically significant (*p* ≤ 0.001). The total biomass values for these 24 genotypes were averaged and illustrated in Fig. 2, representing the difference between averaged biomass distributions across the two years.

We were interested in investigating the relationship between the number of clonemate sporophytes on 6 cm long polyvinyl lines, which we refer as individual counts (Count.Ind), and total biomass on the lines at the time of harvest. For this, we organized the former into 14 groups (Count.Ind = 1, = 2, = 3, …, = 13, >13) and evaluated the distributions of total biomass in these groups (Supplementary Fig. 4A). Qualitatively, the estimated trends showed similar relationships for smaller individual counts. The difference became more pronounced for counts greater than five. In 2019, the trend remained unchanged on average, but decreased when the individual counts exceeded 13. This contrasts with the 2020 trend, where an increase in individual counts leads to a monotonic increase in total biomass. Similarly, we visualized the effect of individual counts on carbon content and nitrogen content (Supplementary Fig. 4B).

To assess the effect of individual counts, as well as the effects of genotype, cross-population, long line ID, and seeding line ID on the phenotypes quantitatively, we utilized a Kruskal–Wallis test (Supplementary Table 4). All the mentioned factors demonstrated statistical significance in both years, except for three cases. The effect of individual counts on carbon content was not statistically significant (H(13) = 12.58, *p* = 0.481). Likewise, the effects of genotype (*H*(344) = 341.60, *p* = 0.526) and cross-population (*H*(3) = 7.55, *p* = 0.056) on nitrogen content were insignificant. Based on these results, we excluded nitrogen content from the downstream analysis.

For the subsequent steps, we obtained best linear unbiased estimates (BLUEs) and best linear unbiased predictions (BLUPs) for total biomass (*n* = 491 and 96 for 2019 and 2020, respectively) and carbon content (*n* = 345). The distributions of BLUEs per cross-population are displayed in Supplementary Fig. 5. Calculating both BLUEs and BLUPs allowed us to perform different estimations of broad-sense heritability (Supplementary Table 5). For total biomass, broad-sense heritability estimates ranged from 0.27 to 0.28 in 2019 and from 0.47 to 0.50 in 2020. For carbon content, broad-sense heritability varied between 0.31 and 0.35.

### Genome-wide association studies for carbon content and total biomass

We screened 378,063 and 379,690 SNPs for carbon content and total biomass, respectively. For carbon content, the first six principal components explained 14% of total variation (9% from the first two), whereas for total biomass, they explained 13% (8.5% from the first two). Out of the total 56 GWAS models, only one model yielded significant results for the former phenotype and three for the latter (Supplementary Table 6). Notably, models that included principal components as covariates to account for population structure yielded no statistically significant associations. At the same time, these models had the most ideal lambda values (≈ 1).

**Fig. 3:**
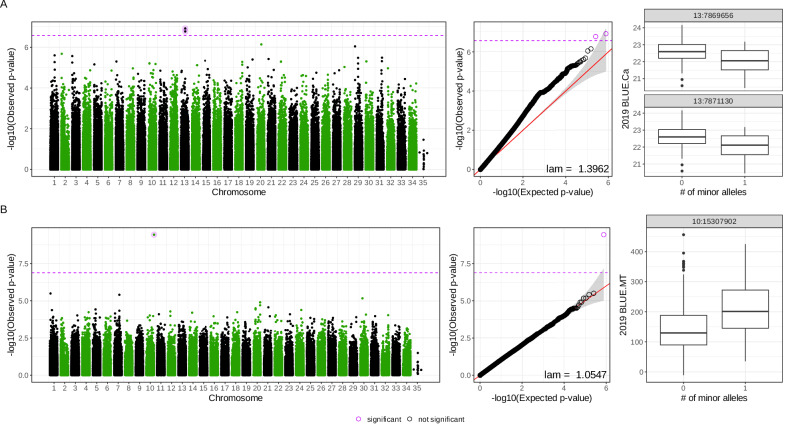
Genome-wide association study results: Manhattan and quantile-quantile plots, BLUE distributions. Manhattan plots, quantile-quantile (QQ) plots, and BLUE distributions for (**A**) carbon content and (**B**) total biomass.

For total biomass, we chose the Blink model, as it had the closest to one genetic inflation factor value among the three models. The results of these models are represented by Manhattan and QQ plots in Fig. 3. We were able to identify two SNPs associated with carbon content and one SNP associated with total biomass (Table 1). Carbon content seemed to be affected negatively by the presence of minor alleles in the identified SNPs, opposite to the total biomass case (Fig. 3). These “negative effect minor alleles” tended to be more abundant in sporophytes from LC-CI and LC-LC cross-populations. The distributions of minor alleles associated with total biomass were slightly different. The majority of them were in sporophytes from LC-CP and LC-LC cross-populations. The range of MAF for all three identified SNPs was between 0.065 and 0.070.

**Table 1:** Summary of the statistically significant SNPs and cross-population contributions to total minor allele numbers.

Phenotype	SNP	MAF	*p*-value	# of minor alleles			
				LC-AQ	LC-CI	LC-CP	LC-LC
BLUE.Ca	13:7869656	0.065	1.24E-07	1	14	5	17
	13:7871130	0.070	1.73E-07	1	17	5	17
BLUE.MT	10:15307902	0.066	3.63E-10	3	1	30	20

*BLUE.Ca* carbon content BLUEs, *BLUE.MT* total biomass BLUEs, *MAF* minor allele frequency.

We identified four relevant genes in total, jgi.p|Macpyr2|9543804 and jgi.p|Macpyr2|9808315 for the carbon content SNPs, jgi.p|Macpyr2|9800916 and jgi.p|Macpyr2|9800917 for the total biomass SNPs (Table 2). According to the annotations, gene products of jgi.p|Macpyr2|9543804, jgi.p|Macpyr2|9808315, and jgi.p|Macpyr2|9800916 were predicted to be glutathione S-transferase, fructose-bisphosphate aldolase, and dynein heavy chain, respectively. For carbon content, the identified genes are involved in carbohydrate transport and metabolism, as well as posttranslational modification. For total biomass, we observed only one annotated gene, whose function was associated with the cytoskeleton. The SNP 13:7869656, located in the coding region of the jgi.p|Macpyr2|9543804 gene, was annotated as a missense variant by SnpEff, leading to an amino acid substitution from threonine to alanine at position 45 of the encoded protein.

**Table 2:** Summary and annotations of genes within a ±10 Kbp window around the statistically significant SNPs.

Phenotype	Gene	Related SNPs	SNPs’ location	Annotations					
				KOG (Description)	KOG (Class)	KOG (Group)	GO (Cellular component)	GO (Biological process)	GO (Molecular function)
BLUE.Ca	jgi.p|Macpyr2|9543804	13:7869656	Coding region	(KOG2903) Predicted glutathione S-transferase	Posttranslational modification, protein turnover, chaperones	CELLULAR PROCESSES AND SIGNALING	-	-	-
		13:7871130	Intronic						
	jgi.p|Macpyr2|9808315	13:7869656	Intergenic (1039 bp upstream)	(KOG1557) Fructose-biphosphate aldolase	Carbohydrate transport and metabolism	METABOLISM	-	(GO:0006096) glycolysis	(GO:0004332) fructose-bisphosphate aldolase activity
		13:7871130	Intergenic (2513 bp upstream)						
BLUE.MT	jgi.p|Macpyr2|9800916	10:15307902	Intronic	(KOG3595) Dyneins, heavy chain	Cytoskeleton	CELLULAR PROCESSES AND SIGNALING	(GO:0030286) dynein complex	(GO:0007018) microtubule-based movement	(GO:0000166) nucleotide binding; (GO:0003777) microtubule motor activity; (GO:0005524) ATP binding; (GO:0016887) ATPase activity; (GO:0017111) nucleoside-triphosphatase activity
	jgi.p|Macpyr2|9800917	10:15307902	Intergenic (5850 bp downstream)	-	-	-	-	-	-

*BLUE.Ca* carbon content BLUEs, *BLUE.MT* total biomass BLUEs, *GO* Gene Ontology database, *KOG* Eukaryotic Orthologous Groups database.

We conducted an additional GWAS analysis for the largest cross-population group, LC-LC (*n* = 270 for total biomass, *n* = 180 for carbon content). Although analyses restricted to common variants (MAF > 0.05) yielded no significant results, inclusion of rarer alleles (0.03 < MAF ≤ 0.05) aimed to identify six additional loci associated with carbon content. Four of them were located near three genes: jgi.p|Macpyr2|8867335, jgi.p|Macpyr2|9809118, and jgi.p|Macpyr2|9809269. The annotations of these genes were limited to Ankyrin (KOG4177) and Amino acid transporter protein (KOG1305).

### Genomic selection models

**Fig. 4:**
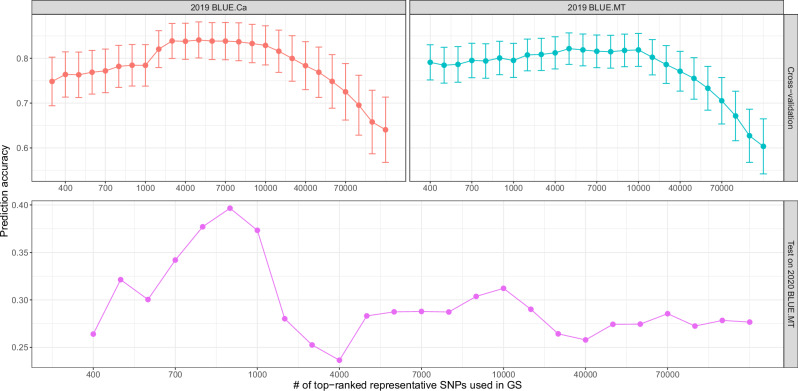
Prediction accuracies (Spearman correlation) for genomic selection (GS) models using different numbers of GWAS-ranked representative SNPs in kinship matrix estimation; the rightmost points correspond to cases where all representative SNPs are used. The upper panels show cross-validation results (mean ± SD) for the 2019 data. The bottom panel illustrates GS models trained on the 2019 BLUE.MT and tested on the 2020 BLUE.MT. BLUE.Ca carbon content BLUEs, BLUE.MT total biomass BLUEs.

Identification of LD-based representative SNPs within chromosomes based on their association with the phenotypes resulted in 94,412 SNPs for carbon content and 94,393 SNPs for total biomass. Using the representative SNPs, we assessed the potential of genomic selection through cross-validation on the 2019 data, as well as tested the genomic selection models on data not used for training (2020 and 2019 data, respectively). Results are illustrated in Fig. 4.

In the case of cross-validation, trends in prediction accuracy for phenotypes were similar when plotted against the number of GWAS-ranked SNPs used. Accuracy initially increased to a certain point, followed by mild fluctuations, and then declined rapidly after using 10,000 representative SNPs in the model. For both phenotypes, the highest mean accuracy was achieved at 5,000 top-ranked representative SNPs, with values of 0.84 for carbon content and 0.82 for total biomass. When actually testing genomic selection models, prediction accuracy was within the 0.23-0.40 interval, with the highest accuracy of 0.396 at 900 top-ranked representative SNPs. The accuracy trend increased until 900 SNPs, then declined and fluctuated around 0.275. Remarkably, when all representative SNPs were used, the prediction accuracy value was 0.277.

## Discussion

### Field performance of farmed crosses

Assessing the field performance of giant kelp sporophytes revealed that phenotypic variation was influenced by both environmental and genetic factors. With respect to survival, genotype losses were minimal compared with sporophyte losses; this, combined with the chi-squared test results, suggests that most mortality was driven by environmental influences (Supplementary Tables 2 and 3). To the best of our knowledge, there are no reported mortality rates specifically for kelp offshore farms; however, we believe our rates are normal based on documented rates from other studies (Seymour et al. 1989; Sogn Andersen et al. 2011; Filbee-Dexter et al. 2020).

Analysis of total biomass between the two years showed no presence of genotype-by-environment interaction. ANOVA results for the shared crosses across the farms indicated that only the main effects (genotype and year) were significant, with the 2020 sporophytes yielding higher biomass. The observed influence of the outplanting period on kelp productivity aligns with previous studies on giant kelp (Stekoll et al. 2021). In our case, the 2019 crosses experienced temperatures higher than those considered natural for growth and development (Harden et al. 2024). Furthermore, the difference between our broad-sense heritability estimates supports this notion. Of note, the heritability estimates were moderate for both farms (~0.27 in 2019; ~0.47 in 2020), underscoring the importance of genetic effects. While we consider the outplantation season to be the main contributor to the environmental component of the observed variation, the season itself encompasses a number of factors directly affecting survival rates and biomass within our genotypes. Among them are interactions with marine bacteria and epiphytes, which can alter community composition and be detrimental to its development and growth, given that giant kelp relies heavily on symbiotic relationships with its microbiome (Michelou et al. 2013; Minich et al. 2018; James et al. 2020). Together, these results signify that while the environment has a substantial impact, genetics also plays a significant role in shaping variation in the traits. Moreover, this variation is detectable and meaningful within our experimental design.

Examining phenotypic variation revealed several key findings. We identified density-dependent effects on nitrogen content and total biomass, with the latter relationships differing between years (Supplementary Table 4 and Supplementary Fig. 4). The observed plateau and subsequent decline in biomass with increasing numbers of clonemates in 2019 likely reflect outplanting season differences in nutrient availability (Zimmerman and Kremer 1984) and increased intraspecific competition for light and space (Graham et al. 2007). In addition, only nitrogen content was not significantly influenced by genotype or cross-population (Supplementary Table 4), which aligns with the fact that it is highly dependent on nutrient availability (Gerard and North 1980; Gerard 1982; Zimmerman and Kremer 1986). Moreover, the kelp-associated microbiome can play a crucial role in nitrogen uptake (Florez et al. 2021; Hochroth and Pfister 2024) by supplying kelp with regenerated forms of nitrogen (Lees et al. 2024).

Overall, these field performance results demonstrate that variation in key production traits, such as biomass and carbon content, is measurable and heritable in realistic farming settings. This is particularly important because biomass is a major lever for reducing kelp production costs. Our results further support genome-driven breeding and selection strategies aimed at increasing biomass and altering carbon content.

### Genotype-phenotype associations for carbon content and total biomass

Genome-wide association studies identified few putative loci associated with carbon content and total biomass, likely due to the polygenic nature of these traits and limited statistical power despite robust genotyping and filtering. We used BLUEs of traits as phenotype values in GWAS because this adjusts genotypic means for environmental effects, leaving only the genotypic signal (Holland and Piepho 2024). Notably, none of the models with significant results were adjusted for population stratification. Adding principal components to the models improved genomic inflation factors but eliminated significant associations. The concentration of minor alleles within specific cross-populations (Table 1) and the correlation between traits and principal components (Supplementary Fig. 6) further suggest that these associations are at least partially confounded by population structure.

Despite these limitations, loci within ~10 Kb of the identified SNPs overlap with genes relevant to this study. Putatively associated genes include fructose-1,6-bisphosphate aldolase (FBA) and glutathione S-transferase (GST) genes for carbon content, and a dynein heavy chain (DHC) gene for total biomass (Table 2). FBA is a key enzyme in glycolysis, gluconeogenesis, and the Calvin cycle (Yang et al. 2023). Its overexpression in plastids positively affects photosynthesis, plant growth, stress tolerance, and carbon metabolism (Uematsu et al. 2012; Cai et al. 2022; Liu et al. 2025). GST is a more general protein with diverse functions (Vaish et al. 2020) and no direct link to carbon metabolism. Even so, studies suggest it may play an intermediate role by altering the carbon metabolite pool (Mueller et al. 2000; Sappl et al. 2009). DHCs are cytoskeletal motor proteins that mediate intracellular transport and mitosis (Roberts et al. 2013; Hou and Witman 2015; Hinchcliffe and Vaughan 2018). Altering these processes may affect growth rate and, consequently, biomass accumulation (Goranov et al. 2009; Weraduwage et al. 2015). While being pertinent, these functional interpretations remain speculative and require validation in other populations or with additional methods. Our attempt to evaluate associations in the largest cross-population group (LC-LC) did not yield significant results until rarer alleles were included; genes associated with significant loci had no direct connection to the traits, according to available annotations.

Altogether, our GWAS results suggest that while detectable genotype-phenotype associations exist for total biomass and carbon content, strong population structure and limited sample sizes constrain statistical power. The identified SNPs and genes should, therefore, be viewed as candidate targets for future validation, including additional experimental steps such as CRISPR/Cas9 or RNAi knockdown, rather than definitive selection targets.

### Efficiency of genomic predictions

Using a moderate number of informative markers yielded accurate phenotype predictions; however, accuracy declined when the number of markers was excessively large. Thus, minimizing redundancy in chromosomes and selecting representative loci are crucial for identifying the top-ranked SNPs and improving prediction accuracy. The observed non-monotonic pattern has also been reported in other taxa (Heinrich et al. 2023), underscoring the need for careful marker selection in genomic selection models. Furthermore, comparing within-year cross-validation with actual testing on two datasets of different origins revealed that the latter achieved its highest accuracy with fewer top-ranked SNPs (5,000 vs 900), suggesting that models suffice a moderate number of markers to generalize.

Counterintuitive at first, this phenomenon is consistent with the curse of dimensionality and overfitting. The effectiveness of distance-based models, including genomic selection, depends on the data’s dimensionality. An increase in the number of dimensions leads to data sparsity, reducing the informativeness of distances between points (Altman and Krzywinski 2018). In SNP data, the more top-related SNPs are used, the less informative genetic relatedness is between two individuals with respect to the trait in question. Because cross-validation evaluates training and testing within the same data distribution, it tends to favor more complex models and overestimate performance, whereas training on one dataset and testing on another emphasizes generalization and limits overfitting.

To sum up, our findings highlight the potential of genomic selection in giant kelp. However, developing breeding models requires careful optimization of a set of loci associated (in a statistical manner) with demanding phenotypes.

### Implications for giant kelp aquaculture in the Southern California Bight

This study has been designed to enable genetics-based breeding programs for giant kelp. Throughout our analyses, we observed that there is a decent degree of heritable variation for key production phenotypes, such as biomass and carbon content, within and between kelp populations. However, consistent with (Johansson et al. 2015), we also observe strong population subdivision and significant phenotypic differences between populations, which align with strong GWAS signals being confounded by population structure. For faster breeding progress, we propose starting with crosses between populations to unlock the most helpful genetic variation. Unfortunately, this conflicts with regulatory limitations, which require only local kelps to be used for local farming and do not permit the production of any F2 individuals from interpopulation crosses.

Overall, the potential for converting kelps into very efficient, robust, and uniform crops appears very high; however, additional strategies, such as careful control of domesticated kelp reproduction (Vissers et al. 2024), must be considered as a part of any breeding program for ocean crops of the future.

## Supplementary information

Supplementary figures

Supplementary tables

## Data Availability

The raw Illumina sequences of 559 gametophytes used to generate the SNP data are available in the NCBI database under the accession code PRJNA1050779 (https://www.ncbi.nlm.nih.gov/sra/PRJNA1050779). Other data and sources supporting the findings are available from the corresponding author upon reasonable request.
