# Supplementary material for: Insights from farming Macrocystis pyrifera offshore: phenotypic analysis, genome-wide association studies, genomic selection
Source: Heredity (Edinb). 2026 Jun 18;135(7):486–96. doi: 10.1038/s41437-026-00855-4 (PMC13451209; doi:10.1038/s41437-026-00855-4)
Supplement: Supplementary file 1 [file 41437_2026_855_MOESM1_ESM.docx]

**Supplementary Figure 1.** Visualizations of the crossing designs for the 2019 and 2020 giant kelp farms. Total number of outplanted sporophytes is 2500 (500 sporophyte genotypes x 5 replicates) and 960 (96 sporophyte genotypes x 10 replicates) for the 2019 and 2020 farms, respectively. AQ – Arroyo Quemado; CI – Catalina Island; CP – Camp Pendleton; LC – Leo Carrillo.

**Supplementary Figure 2.** Analysis of the environmental variables recorded for the 2019 and 2020 farms.

**Supplementary Figure 3.** Correlation analysis of the phenotypes measured for the 2019 and 2020 farms. ns – not significant; * – p-value ≤ 0.05; ** – p-value ≤ 0.01; *** – p-value ≤ 0.001; **** – p-value ≤ 0.0001.

**Supplementary Figure 4.** Distributions of (A) total biomass, (B) carbon content, and nitrogen content relative to individual counts (the number of clonemate sporophytes on a polyvinyl line). The red line shows the trends in phenotypes across individual counts formed as groups.

**Supplementary Figure 5.** Distributions of the phenotypes’ BLUEs across different cross-populations.

**Supplementary Figure 6.** Relationships between principal components and BLUEs for 2019 total biomass and carbon content.
