# Supplementary material for: Insights from farming Macrocystis pyrifera offshore: phenotypic analysis, genome-wide association studies, genomic selection
Source: Heredity (Edinb). 2026 Jun 18;135(7):486–96. doi: 10.1038/s41437-026-00855-4 (PMC13451209; doi:10.1038/s41437-026-00855-4)
Supplement: Supplementary file 2 [file 41437_2026_855_MOESM2_ESM.docx]

| Phenotypes | Common | Count.Ind | Number of clonemates (individual sporophytes per replicate on a polyvinyl string) |
| --- | --- | --- | --- |
|  |  | Mass.Stipe | Wet mass in grams of stipes and pneumatocysts (no blades or holdfasts) from all clonemates in a replicate |
|  |  | Mass.Blade | Wet mass in grams of blades from all clonemates in a replicate |
|  |  | Mass.Total | Sum of stipe and blade biomass in grams |
|  | 2019 | Count.Blade | Number of blades counted |
|  |  | Carbon | Carbon content, % of dry mass |
|  |  | Nitrogen | Nitrogen content, % of dry mass |
| Variables | Common | Pop.Male | Population of male gametophyte |
|  |  | Pop.Female | Population of female gametophyte |
|  |  | Pop.Cross | Cross-population; formed as Pop.Male-Pop.Female (for example, LC-AQ) |
|  |  | Date.Plant | Date sporophytes were outplanted |
|  |  | Date.Harv; Date.Obs | Date sporophytes were harvested (for 2019 phenotypes were observed at the same day) |
|  |  | Line.Long | Long line ID |
|  |  | Line.Seed | Seeding line ID |
|  | 2019 | Replicate | The replicate ID of a cross |

**Supplementary Table 1.** Description of the phenotypes and environmental variables.

| **Year** | **Phenotype** | **Total** | | **Survived** | | **Survived, %** | | **Phenotyped** | | **Phenotyped, %** | |
| --- | --- | --- | --- | --- | --- | --- | --- | --- | --- | --- | --- |
|  |  | **Spo** | **Gen** | **Spo** | **Gen** | **Spo** | **Gen** | **Spo** | **Gen** | **Spo** | **Gen** |
| 2019 | Mass.Total | 2500 | 500 | 1686 | 491 | 67.44 | 98.2 | 1669 | 491 | 66.76 | 98.2 |
|  | Carbon |  |  |  |  |  |  | 1269 | 345 | 50.76 | 69 |
|  | Nitrogen |  |  |  |  |  |  | 1267 | 345 | 50.68 | 69 |
| 2020 | Mass.Total | 960 | 96 | 627 | 96 | 65.31 | 100 | 614 | 96 | 63.96 | 100 |

**Supplementary Table 2.** Description of the phenotypes’ dynamics from outplanting to phenotyping. Spo – sporophytes; Gen – genotypes.

| **Variable** | **Farm** | **N** | **Df** | **χ2-statistic** | **p-value** |
| --- | --- | --- | --- | --- | --- |
| Genotype | 2019 | 2500 | 499 | 654.70 | 3.19E-06 |
|  | 2020 | 960 | 95 | 178.28 | 5.01E-07 |
| Pop.Cross | 2019 | 2500 | 3 | 32.54 | 4.03E-07 |
|  | 2020 | 960 | 15 | 58.89 | 3.92E-07 |
| Line.Long | 2019 | 2500 | 9 | 337.59 | 2.71E-67 |
|  | 2020 | 960 | 3 | 2.48 | ***4.78E-01*** |
| Line.Seed | 2019 | 2500 | 49 | 408.05 | 4.20E-58 |
|  | 2020 | 960 | 19 | 51.89 | 6.87E-05 |

**Supplementary Table 3.** Chi-square tests of independence between sporophyte survival and selected environmental variables, including genotypes. N – number of datapoints; Df – degrees of freedom; bold italics indicate insignificant results (p-value > 0.05).

| **Phenotype** | **Farm** | **Variable** | **N** | **Df** | **H-statistic** | **p-value** |
| --- | --- | --- | --- | --- | --- | --- |
| Mass.Total | 2019 | Genotype | 1669 | 490 | 741.39 | 1.45E-12 |
|  |  | Pop.Cross |  | 3 | 32.70 | 3.73E-07 |
|  |  | Line.Long |  | 9 | 67.78 | 4.15E-11 |
|  |  | Line.Seed |  | 49 | 113.85 | 4.47E-07 |
|  |  | CI.Group |  | 13 | 426.21 | 6.45E-83 |
|  | 2020 | Genotype | 614 | 95 | 180.43 | 2.95E-07 |
|  |  | Pop.Cross |  | 15 | 70.67 | 3.39E-09 |
|  |  | Line.Long |  | 3 | 16.99 | 7.10E-04 |
|  |  | Line.Seed |  | 19 | 102.36 | 2.00E-13 |
|  |  | CI.Group |  | 13 | 180.53 | 1.32E-31 |
| Carbon | 2019 | Genotype | 1269 | 344 | 430.34 | 1.05E-03 |
|  |  | Pop.Cross |  | 3 | 26.58 | 7.21E-06 |
|  |  | Line.Long |  | 9 | 97.31 | 5.49E-17 |
|  |  | Line.Seed |  | 49 | 182.21 | 3.24E-17 |
|  |  | CI.Group |  | 13 | 12.58 | ***4.81E-01*** |
| Nitrogen | 2019 | Genotype | 1267 | 344 | 341.60 | ***5.26E-01*** |
|  |  | Pop.Cross |  | 3 | 7.55 | ***5.62E-02*** |
|  |  | Line.Long |  | 9 | 130.82 | 8.05E-24 |
|  |  | Line.Seed |  | 49 | 204.11 | 7.90E-21 |
|  |  | CI.Group |  | 13 | 35.45 | 7.23E-04 |

**Supplementary Table 4.** Testing the influence of sporophyte genotypes and specific environmental variables on phenotypic variance using the Kruskal-Wallis test. N – number of datapoints; Df – degrees of freedom; bold italics indicate insignificant results (p-value > 0.05).

| **Phenotype** | **Farm** | **Broad-sense heritability (H^2^)** | | |
| --- | --- | --- | --- | --- |
|  |  | **Holland’s** | **Regression-based** | **Shrinkage-based** |
| Mass.Total | 2019 | 0.282 | 0.270 | 0.270 |
|  | 2020 | 0.471 | 0.496 | 0.488 |
| Carbon | 2019 | 0.309 | 0.341 | 0.348 |

**Supplementary Table 5.** Broad-sense heritability estimates for the selected phenotypes.

| **Phenotype** | **Model** | **# of used PCs** | **# of significant SNPs** | **Lambda** | **Rank** |
| --- | --- | --- | --- | --- | --- |
| BLUE.Ca | GLM | 0 | 2 | 1.3962 | 1 |
| BLUE.MT | Blink | 0 | 1 | 1.0547 | 1 |
|  | FarmCPU | 0 | 12 | 0.9497 | 2 |
|  | GLM | 0 | 1 | 1.4342 | 3 |

**Supplementary Table 6.** Summary of successful GWAS models for each phenotype, ranked by Lambda. BLUE.Ca – carbon content BLUEs; BLUE.MT – total biomass BLUEs; Lambda – genetic inflation factor.
